# Prevalence and determinants of hypertension in underrepresented indigenous populations of Nepal

**DOI:** 10.1371/journal.pgph.0000133

**Published:** 2022-02-18

**Authors:** Tsedenia Workneh Denekew, Yoshina Gautam, Dinesh Bhandari, Guru Prasad Gautam, Jeevan Bahadur Sherchand, Amod K. Pokhrel, Aashish R. Jha

**Affiliations:** 1 Genetic Heritage Group, Program in Biology, New York University Abu Dhabi, Abu Dhabi, United Arab Emirates; 2 Himalayan Diversity Project, Department of Biomedical Data Science, Stanford University, Stanford, Palo Alto, United States of America; 3 School of Public Health, University of Adelaide, Adelaide, Australia; 4 Public Health Research Lab, Tribhuvan University Institute of Medicine, Maharajgunj, Nepal; 5 Department of Geography, Tribhuvan University, Nepalgunj, Nepal; 6 Society for Legal and Environmental Analysis and Development Research, Kathmandu, Nepal; 7 On-Campus/On-Line MPH program, School of Public Health, University of California, Berkeley, CA, United States of America; Augusta University, UNITED STATES

## Abstract

Indigenous populations residing in low- and middle-income countries (LMICs) are highly underrepresented in medicine and public health research. Specifically, data on non-communicable diseases (NCDs) from indigenous populations remains scarce. Despite the increasing burden of NCDs in the Himalayan region, their prevalence in many indigenous populations remains understudied. The nationally representative public health surveys often do not include the indigenous communities, especially those that reside in rural areas or exist in small numbers. This observational cross-sectional survey study aimed to assess the prevalence of three NCD risk factors namely obesity, hypertension, and tachycardia and identify dietary and lifestyle variables associated with them across underrepresented indigenous populations of Nepal. A total of 311 individuals (53.3% women, 46.7% men) with mean age 43±15 years from 12 indigenous Nepali communities residing in rural (47.9%) or semi-urban (52.1%) areas volunteered to participate in this study. Univariate tests and multivariable logistic regressions were used to analyze the survey data. The mean systolic and diastolic blood pressures were 121.3±19.5 mmHg and 81.3±11.8 mmHg respectively. Overall, the prevalence of obesity and tachycardia was low (0.64% and 3.22%, respectively) but hypertension was prevalent at 23.8%. Hypertension was not significantly different across populations, but it was associated with age, BMI, and tobacco use, and collectively, these variables explained 13.9% variation in hypertension prevalence. Although we were unable to detect direct associations between individual determinants of hypertension identified in non-indigenous Nepalis, such as education levels, alcohol consumption, and smoking in this study, having one or more determinants increased the odds of hypertension in the indigenous participants. Furthermore, ~14% of the hypertensive individuals had none of the universally identified hypertension risk factors. The lack of association between previously identified risk factors for hypertension in these individuals indicates that the additional determinants of hypertension remain to be identified in indigenous Nepali populations.

## Introduction

Non-communicable diseases (NCDs) are the leading cause of morbidity and mortality in the world [1]. The global non-communicable death burden currently stands at 71%, with 41 million out of the world’s 57 million deaths linked directly to NCDs [2]. Although communicable diseases still pose a major threat in low- and middle-income countries (LMICs), they are also witnessing a steady increase in the incidence of NCDs. The proportion of deaths due to NCDs has already surpassed communicable diseases in these nations [3–5]. The vast majority of NCD related deaths (78%) occur in LMICs [1], indicating that the existing healthcare infrastructures in LMICs are currently poorly equipped to tackle the increasing burden of NCDs. Despite growing awareness of high NCD burden, research designed to understand their prevalence and determinants within these regions is minimal [3]. Indigenous populations are even more underrepresented in medical research from LMICs as nationally representative public health studies designed to reflect the overall public health of a nationwide population do not include all ethnic communities, especially those that reside in rural areas or exist in small numbers. Therefore, the prevalence of NCDs and their determinants in indigenous populations remains obscure [6–8].

The Himalaya consists of three ecological regions: high mountains with elevation over 1,500 meters above the sea levels (masl), hills with elevation 600–1,500 masl, and the Terai plains (<600 masl). Nepal is a Himalayan country with 29 million people comprising over 120 ethnolinguistic groups. Fifty-nine of these groups are originally recognized as “indigenous” by the Government of Nepal [9, 10] and several groups in Terai are collectively referred to as “Dalit”. A majority (>90%) of the population reside in the hill and Terai regions and many ethnic groups have historically localized to certain geographical areas, except a handful of urban cities where recent migration has led to a mix of co-residing populations of diverse ethnicities. A recent nationally representative cross-sectional survey in Nepal showed that NCDs such as diabetes, obesity, and hypertension are highly prevalent in the general population [11–15]. An alarming 26% of the general population in Nepal had hypertension (systolic blood pressure SBP ≥ 140mmHg or diastolic blood pressure DBP ≥ 90) and 21% of the adult population were overweight or obese (BMI ≥ 25). Almost all of the 4,200 respondents (99.6%) showed at least one previously established NCD associated major risk factors such as smoking, harmful use of alcohol, consumption of low fiber diet rich in salt and sugar, physical inactivity, overweightness/obesity, raised blood pressure, elevated blood glucose, and raised total cholesterol, underscoring a looming health crisis that the country’s health infrastructure is unable to handle [11]. Other nationally representative surveys from Nepal have also highlighted the link between economic status and hypertension and called for exploration of socio-economic status and disease burden [13]. Indigenous populations are among the most marginalized and economically challenged in Nepal [13]. However, due to their small population sizes and lack of accessibility, many of the Nepali indigenous populations have been underrepresented in nationally representative surveys. When included, individuals from several ethnically distinct Nepali indigenous groups with unique traditions and lifestyles have been grouped into a single category called “*janajatis*” [16]. Since they make up a third of the country’s population, understanding the prevalence of NCDs and their determinants in indigenous Nepali groups may provide new insights in tackling the increasing burden of NCDs in the nation as a whole [7].

This study aims to assess the prevalence of NCDs and their determinants in diverse underrepresented ethnic groups in Nepal. We found that NCDs such as hypertension is prevalent at appreciable frequencies in indigenous Nepali populations but its determinants in indigenous populations may differ from non-Indigenous Nepalis. Our results may help policymakers and public health officials develop preventative approaches to address the increasing burden of NCDs in these communities.

## Materials and methods

### Ethical considerations

This study is a part of a multifaceted study that was approved by the Stanford University Institutional Review Board (IRB) as well as the Ethical Review Board of Nepal Health Research Council (NHRC).

### Study design

This population-based cross-sectional study was conducted to evaluate the prevalence of non-communicable diseases and associated risk factors in diverse indigenous populations of Nepal. The study was conducted from February—May 2016. Individuals from 12 different ethnic groups residing in the hills and Terai plains of Nepal were recruited via household visits. Among these, 11 groups are officially recognized as “indigenous” by the Government of Nepal [9] and they include Bote, Chepang, Darai, Kusunda, Maajhi, Newar, Raji, Raute (Dadeldhura), Tamang, Thami, and Tharu. The Musahars are officially classified as “Dalit” and are known to be native inhabitants of the Terai plains [17]. Within each ethnic group, male and female adults over 18 years old whose parents and grandparents were reported to be from the same ethnic group (non-multiracial) were invited to participate via signing an informed consent form. Unrelated individuals, i.e. individuals who did not share a grandparent, were randomly selected from the community to participate in this study and only one individual per household was recruited. A total of 337 individuals aged 18–83 volunteered to participate in this study. Children, minors under the age of 18, and pregnant women were excluded from participating in this study.

### Survey data collection

Demographic, anthropogenic, environmental, and dietary data were obtained from the participants using a survey questionnaire by a trained enumerator (YG). The survey questionnaire was aligned with WHO STEPS Instrument with some modifications to reflect the traditional Nepali lifestyles. The survey questionnaire included demographic variables such as age, gender, education level, and marital status, diet and physical activity, medical histories, and behavioral practices such as smoking, use of non-smoking tobacco products (chewing tobacco), and alcohol consumption, along with several environmental variables such as area of residence, type of cooking fuel used in home, source of drinking water, etc. (S1 Table). Participants’ responses to the relevant survey data questionnaires are included in (S2 Table).

### Phenotypic measurements

Trained enumerator measured systolic and diastolic blood pressures and heart rates using an automatic blood pressure monitor (Omron, BP791IT). Participants were seated comfortably, and four readings of blood pressure were recorded at 3-minute intervals. The last three readings were averaged for subsequent analysis. Participants with systolic blood pressure (SBP) ≥ 140 mmHg and/or diastolic blood pressure (DBP) ≥ 90 mmHg were considered hypertensive. Systolic and diastolic hypertension were defined separately as ≥ 140mmHg and ≥ 90mmHg, respectively. Weight was measured using a balanced scale (Seca 869). The scale was placed on a flat surface and legs were adjusted until the indicator level was at the center position. Height was measured while the participants were standing on the balanced weighing scale. Height and weight were measured three times and averaged. Body mass index (BMI) was calculated as averaged weight in kilograms divided by the square of averaged height in meters. Participants with BMI < 18.5 kg/m^2^ were considered underweight and those with BMI 18.5–24.9kg/m^2^ were considered normal. Participants with BMI ≥ 25 kg/m^2^ and ≥ 30 kg/m^2^ were considered overweight and obese, respectively.

### Statistical analysis

All statistical analyses were performed using STATA (version 16.0) and visualized using R (version 3.6.1). Fifty variables were initially obtained from the survey questionnaire, and 29 variables with >95% identical answers across the dataset were removed from subsequent analyses. Summary statistics were calculated and associations between categorical variables were first assessed using a chi-squared test. Individuals were classified as hypertensive (SBP ≥ 140 mmHg and/or diastolic blood DBP ≥ 90 mmHg) and non-hypertensive (SBP < 140 mmHg and diastolic blood DBP < 90 mmHg) using binary categorization (0 = non-hypertensive and 1 = hypertensive), which was used as dependent variable for calculating odds ratios. Associations between factors and hypertension were assessed by performing univariate and multivariable logistic regressions and crude and adjusted odds ratios were calculated. To perform logistic regression, the continuous variables—BMI, age, and household size–were classified into groups of equal proportions. These analyses were performed separately to identify risk factors associated with systolic hypertension (SBP ≥ 140 mmHg) and diastolic hypertension (DBP ≥ 90 mmHg). To assess associations between the previously established determinants and hypertension, we first created a new variable by counting the total number of the five previously established determinants in each participant such that each participant had a score between 0 and 5. Only 3 individuals had 4 risk factors and none had 5. Due to limited numbers of individuals with 4 and 5 risk factors, individuals with 3 or more risk factors were pooled together to create four categories (0, 1, 2, or ≥3). Next, we compared whether including this variable improved the fit compared to a null model (with no variables) using a Wald test. Finally, we used a logistic regression to assess the odds ratio for hypertension with increasing number of risk factors (0 vs 1, 2, or ≥3). Statistical tests with P-value < 0.05 were considered significant.

## Results

### Population characteristics

A total of 337 individuals were recruited, of which 26 individuals had incomplete information and were removed from subsequent analyses, resulting in 311 participants. These participants belonged to 12 populations of which 11 are officially recognized as “indigenous” by the Government of Nepal [9] and the Musahar, known to be natives of the Terai plains [17], are officially classified as Dalit. The populations included in this study are described in Table 1.

**Table 1:** Populations, census sizes, sampling locations and status of the participating populations.

Populations	Census size (26,494,504)	Official Status	Economic Status	Sample size (311)	Sample location (District and Provinces)
Tharu	1,737,470	Indigenous	Marginalized	84	Sarlahi, Province 2, Dhangadhi, Sudurpaschim Province, and Chitwan, Bagmati Province
Tamang	1,539,830	Indigenous	Marginalized	13	Dolakha, Bagmati Province,
Newar	1,321,933	Indigenous	Advanced	53	Lalitpur and Makwanpur,Bagmati Province
Musahar	234,490	Dalit	Not Available	18	Chitwan, Bagmati Province and Sarlahi, Province 2
Maajhi	83,727	Indigenous	Highly marginalized	31	Sindhupalchowk, Bagmati Province
Chepang	68,399	Indigenous	Highly marginalized	27	Chitwan, Bagmati Province
Thami	28,671	Indigenous	Highly marginalized	13	Dolakha, Bagmati Province
Darai	16,789	Indigenous	Marginalized	16	Chitwan, Bagmati Province
Bote	10,397	Indigenous	Highly marginalized	6	Chitwan, Bagmati Province
Raji	4,235	Indigenous	Endangered	21	Bardiya, Lumbini Province
Raute	618	Indigenous	Endangered	24	Dadeldhura, Sudurpaschim Province
Kusunda	273	Indigenous	Endangered	3	Dang, Lumbini Province
Other	--	--	--	2	--

The populations included in this study, their census sizes based on the 2011 census [18], sample size in this study, and sampling locations are shown. Official recognition indicates whether the populations are recognized as “indigenous” by the Government of Nepal [9]. Economic status reflects the categorization of these populations based on their development indicators by Nepal Federation of Indigenous Nationalities (NEFIN) [9]. Other: individuals of mixed ancestries.

The social and demographic characteristics of the participating populations are shown in Table 2. Of the 311 participants, 53.4% were from the Hills, and 46.6% were from the Terai. Participants were recruited from 12 indigenous groups residing in rural (47.9%) or semi-urban (52.1%) areas, and groups with less than 10 participants (Kusunda and Bote) were grouped as “other.” About half (53.3%) of the participants were females. Participants were on average 43±15 years old with 43.7%, 40.2%, and 16% between the ages of 18–39, 40–61, and 61–83 years. Half of the participants had no formal schooling, and another 21% had only completed primary school. A vast majority of participants (88%) did not access the internet. Most (97%) of participants consumed fresh vegetables twice a day (S2 Table), which is consistent with previous observations in Nepal [19]. Consistent with their agrarian lifestyle, a majority of participants were physically active on a regular basis (80.7%). Animal protein (e.g., fish, meat, and dairy) was rare in their diet and 67.5% of the study participants ate home cooked meals on a daily basis, and a vast majority of participants consumed fermented foods on a regular basis (91%). About half of the participants (48.6%) responded yes when asked if they perceived scarcity of food within their respective households. Smoking and use of non-smoking (chewing) tobacco products were observed in 24% and 22% of participants, respectively, and 47% of participants consumed some level of alcohol on a daily or weekly basis.

**Table 2:** Socio-demographic and dietary factors in the study participants.

Variables	N (n = 311)	Percent Prevalence	Percent Prevalence by gender	
**Age**		Overall %	Male % (n = 145)	Female %(n = 166)
18–39	136	43.73	33.10	53.01
40–61	125	40.19	46.20	34.94
62–83	50	16.08	20.68	12.05
**BMI**				
Underweight (<18.5)	54	17.36	17.93	16.87
Normal (18.5–25)	208	66.88	61.37	71.68
Overweight/Obese (> = 25)	49	15.76	20.68	11.44
**Education**				
Never/Rarely formal education	163	52.41	42.06	61.44
Primary Education	65	20.9	26.89	15.66
Secondary Education	58	18.65	21.38	16.26
Post-Secondary	25	8.04	9.60	6.60
**Internet Access**				
Never/Rarely	274	88.1	86.20	89.76
Weekly/Daily	37	11.9	13.79	10.24
**Tobacco Use**				
Never/Rarely	244	78.46	60.68	93.97
Weekly/Daily	67	21.54	39.31	6.02
**Smoking**				
Never/Rarely	234	75.24	63.44	85.54
Weekly/Daily	77	24.76	36.55	14.46
**Alcohol Drinking**				
Never/Rarely	164	52.73	40.68	63.25
Weekly/Daily	147	47.27	59.31	36.75
**Exercise**				
Never/Rarely	60	19.29	22.07	16.68
Weekly/Daily	251	80.71	77.93	83.13
**Location**				
Semi urban	162	52.09	53.79	50.60
Rural	149	47.91	46.21	49.39
**Geography**				
Hills	166	53.38	60.68	46.98
Terrai	145	46.62	39.31	53.01
**Household size**				
(1–4)	93	29.9	28.27	31.32
(5–9)	183	58.84	55.86	61.44
(10+)	35	11.25	15.86	7.23
**Altitude**				
<500	169	54.34	46.89	60.84
[500–1500]	105	33.76	38.62	29.52
>1500	37	11.9	14.48	9.64
**Milk**				
Never/Rarely	277	89.07	88.97	89.16
Weekly/Daily	34	10.93	11.03	10.84
**Yoghurt**				
Never/Rarely	259	83.28	85.52	81.32
Weekly/Daily	52	16.72	14.48	18.67
**Fermented food**				
Never/Rarely	27	8.68	12.41	5.40
Weekly/Daily	284	91.32	87.58	94.58
**Cooked food**				
Never/Rarely	101	32.48	48.27	18.67
Weekly/Daily	210	67.52	51.72	81.32
**Scarcity of food**				
No	160	51.45	55.17	48.19
Yes	151	48.55	44.80	51.80
**Fish**				
Never/Rarely	277	89.07	85.52	92.16
Weekly/Daily	34	10.93	14.48	7.83
**Meat**				
Never/Rarely	220	70.74	68.27	72.89
Weekly/Daily	91	29.26	31.72	27.11

A total of 311 participants comprising of males and females were included in this study. Column 1 describes categorization of participants into groups. BMI: Body Mass Index; Education: participants with no schooling (Never/Rarely formal education), Primary Education: grades 1–5; Secondary Education: grades 5–10; Post-secondary education: grade 11 or higher. Altitude in meters above the sea level.

### Prevalence of non-communicable diseases

**Fig 1:**
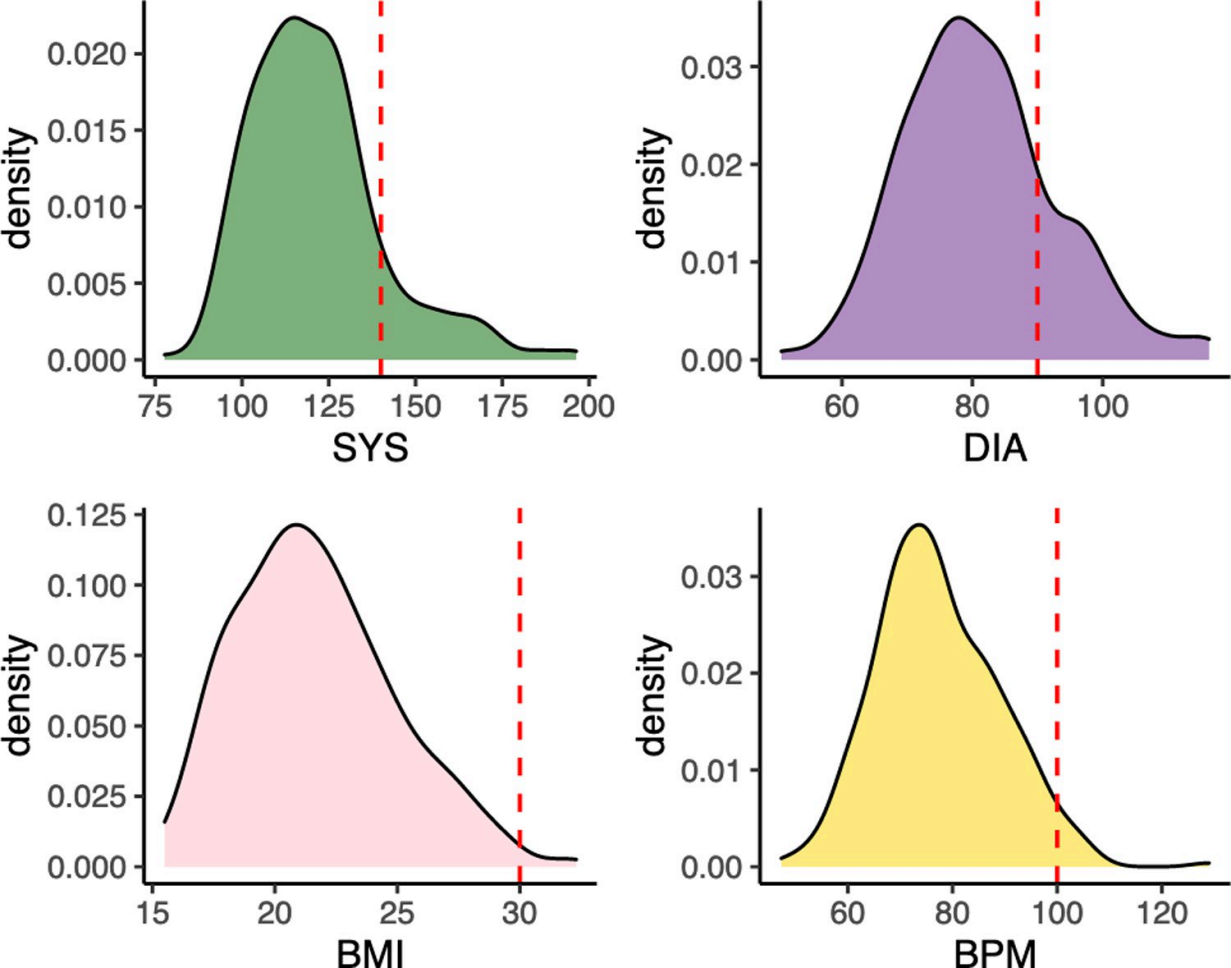
Prevalence of Non-Communicable Disease (NCD) risk factors in indigenous peoples of Nepal. Density plots showing the distributions of the four NCD risk factors measured from 311 Nepali individuals. Clockwise from the top: systolic blood pressure (SBP), diastolic blood pressure (DBP), heart rate measured as beats per minute (BPM), body mass index (BMI). Dotted lines indicate threshold for disease status: SBP = 140 mmHG, DBP = 90 mmHG, BMI = 30, and heart rate = 100 BPM.

We assessed blood pressure, body mass index (BMI), and heart rate in these participants to determine the prevalence of hypertension, obesity, and tachycardia (Fig 1). The mean systolic and diastolic pressures were 121.3±19.5 mmHg and 81.3±11.8 mmHg respectively. Overall, the prevalence of hypertension, defined as SBP ≥ 140 mmHg and/or DBP ≥ 90 mmHg, was 23.8%. The prevalence of hypertension was 27.7% and 19.3% in the Hills and Terai respectively. Hypertension was more prevalent among men (31.7%) than women (16.8%, P = 0.003, *chi-square test*). On average, women had lower systolic and diastolic blood pressures (n = 166, mean SBP = 115.5±17.9 mmHg and mean DBP = 78.9±11.3 mmHg) compared to men (n = 145, mean SBP = 127.0±19 mmHg and mean DBP = 84.0±11.7 mmHg, P = 0.049 and 0.003 for SBP and DBP respectively, *Student’s t test*). The average BMI and heart rate in the overall dataset were 21.6±3.2 and 77.3±11.7 bpm (Fig 1). About 15% (N = 47) of participants were overweight, while both obesity (BMI>30) and tachycardia (heart rate >100 bpm) were negligible (N = 2 and 10, respectively). Because of its higher observed prevalence in these participants, we focused on hypertension in subsequent analyses.

### Hypertension associated risk factors

Several modifiable risk factors such as higher BMI, alcohol consumption, smoking, lack of physical activity, and a processed diet rich in sodium and carbohydrates are classic determinants of hypertension [20]. We sought to identify whether these and other factors are associated with hypertension in our participants. We evaluated the association between hypertension, which was defined as SBP ≥ 140 mmHg and/or diastolic blood DBP ≥ 90 mmHg, and 19 factors using a univariate logistic regression (Table 3). This analysis revealed age, BMI, sex, chewing tobacco use and milk consumption was associated with hypertension in these participants (P<0.05, Table 3). Complex traits such as hypertension can be influenced by more than one variable and some of these variables may be correlated with one another. To account for interdependence between variables, we next performed a multivariable logistic regression and calculated the adjusted Odds Ratio. This analysis revealed that age, use of non-smoking tobacco products, and BMI were significantly associated with hypertension (P<0.05, Table 3). Compared to participants aged 18–39, individuals within the age range 40–61 and 62–83 were more likely to develop hypertension (adjusted OR = 2.8, 95% CI: 1.3–6.2 and 7.6, 95% CI: 2.8–20.8 respectively). We did not find significant associations between smoking and hypertension but compared to those who do not use non-smoking (chewing) tobacco products (N = 244), chewing tobacco users (N = 67) were more likely to develop hypertension (adjusted OR = 2.5, 95% CI:1.1–5.6). Furthermore, participants whose diet includes milk on a weekly or daily basis (n = 34) had higher chances of developing hypertension relative to individuals who rarely or never consumed dairy products (adjusted O.R = 3.6, 95% CI: 1.3–10.1). Finally, we performed a linear multivariable regression analysis using hypertension as the dependent variable and the 19 variables as independent variables. This model explained ~14% of the variance in hypertension in these populations (df = 24, R^2^ = 0.21; adjusted R^2^ = 0.139), indicating that additional determinants of hypertension remain to be identified in these populations.

**Table 3:** Factors associated with hypertension in study participants.

	UNIVARIATE LOGISTIC REGRESSION					MULTIVARIABLE LOGISTIC REGRESSION		
Hypertension	Crude Odds Ratio	[95%Conf.Interval]		P value	Adjusted Odds Ratio	[95%Conf.Interval]		P value
**Age**								
18–39	1				1			
**40–61**	**3.263**	**1.684**	**6.323**	**0.000**	**2.872**	**1.324**	**6.232**	**0.008**
**62–83**	**6.872**	**3.173**	**14.882**	**0.000**	**7.668**	**2.826**	**20.805**	**0.000**
**BMI**								
Underweight (<18.5)	1				1			
Normal (18.5–25)	1.679	0.741	3.804	0.215	2.134	0.825	5.523	0.118
**Overweight/Obese (> = 25)**	**3.642**	**1.415**	**9.374**	**0.007**	**4.048**	**1.234**	**13.272**	**0.021**
**Sex**				** **				** **
Male	1				1			
**Female**	**0.437**	**0.255**	**0.746**	**0.002**	0.765	0.354	1.651	0.495
**Education**								
Never/Rarely formal education	1				1			
Primary Education	1.474	0.791	2.746	0.222	1.571	0.703	3.51	0.271
Secondary Education	0.461	0.202	1.052	0.066	0.753	0.26	2.183	0.601
Post-Secondary	0.251	0.057	1.108	0.068	0.816	0.113	5.879	0.84
**Internet Access**								
Never/Rarely	1				1			
Weekly/Daily	0.353	0.121	1.033	0.057	0.361	0.081	1.616	0.183
**Tobacco Use**								
Never/Rarely	1				1			
**Weekly/Daily**	**2.369**	**1.319**	**4.256**	**0.004**	**2.492**	**1.102**	**5.631**	**0.028**
**Smoking**								
Never/Rarely	1				1			
Weekly/Daily	1.066	0.585	1.942	0.834	0.667	0.317	1.399	0.283
**Alcohol Drinking**								
Never/Rarely	1				1			
Weekly/Daily	**2.054**	**1.205**	**3.499**	**0.008**	1.588	0.812	3.104	0.176
**Exercise**								
Never/Rarely	1				1			
Weekly/Daily	0.606	0.326	1.126	0.113	0.546	0.265	1.195	0.135
**Location**								
Semi urban	1				1			
Rural	1.04	0.617	1.753	0.884	1.102	0.538	2.295	0.776
**Geography**								
Hills	1				1			
Terrai	0.624	0.366	1.065	0.084	0.645	0.351	1.887	0.632
**Household size**								
(1–4)	1				1			
(5–9)	1.053	0.581	1.908	0.865	1.131	0.578	2.331	0.675
(10+)	1.371	0.569	3.306	0.482	0.691	0.239	2.366	0.627
**Milk**								
Never/Rarely	1							
Weekly/Daily	**2.192**	**1.038**	**4.63**	**0.040**	**3.597**	**1.333**	**10.139**	**0.012**
**Yoghurt**								
Never/Rarely	1							
Weekly/Daily	0.835	0.405	1.721	0.624	0.461	0.179	1.239	0.127
**Fermented food**								
Never/Rarely	1				1			
Weekly/Daily	0.495	0.216	1.133	0.096	1.237	0.685	2.436	0.429
**Cooked food**								
Never/Rarely	1				1			
Weekly/Daily	0.855	0.493	1.482	0.576	0.697	0.349	1.53	0.405
**Scarcity of food**								
No	1				1			
Yes	1.541	0.911	2.608	0.107	1.406	0.742	2.82	0.278
**Fish**								
Never/Rarely	1				1			
Weekly/Daily	1.174	0.522	2.642	0.698	1.594	0.504	4.272	0.482
**Meat**								
Never/Rarely	1				1			
Weekly/Daily	1.553	0.893	2.701	0.119	1.719	0.834	3.521	0.143
**Constant**					0.07	0.008	0.353	0.002

Hypertension was defined as systolic blood pressure ≥ 140 mmHg and/or diastolic blood pressure ≥ 90 mmHg. Column 1 includes variables tested for association with hypertension using univariate and multivariable logistic regressions in the 311 study participants from Nepal. Crude odds ratio was derived from the univariate logistic regression and adjusted odds ratio was derived from the multivariable logistic regression. Significant results with p values < 0.05 are shown in bold.

We repeated this analysis separately for systolic and diastolic hypertension. Univariate logistic regression analyses revealed that systolic hypertension was associated with age, sex, milk consumption and diastolic hypertension was associated with age, BMI, sex, tobacco use, alcohol and meat consumptions (Tables 4 and 5). After accounting for multiple confounding variables using multivariable logistic regressions, we found that milk consumption was associated with systolic hypertension (adjusted OR of 4.3, 95% CI: 1.3–15.1, Table 4) and meat consumption was associated with diastolic hypertension (adjusted OR of 2.2, 95% CI: 1.1–4.6, Table 5). Individuals consuming milk and meat frequently had higher chances of developing systolic and diastolic hypertension respectively. However, these correlations should be interpreted with caution as further investigations with larger cohorts supplemented with molecular markers are needed to identify causal factors in these populations.

**Table 4:** Factors associated with systolic hypertension in study participants.

	UNIVARIATE ANALYSIS				MULTIVARIATE ANALYSIS			
	CrudeOdds Ratio	[95%Conf.Interval]		P value	AdjustedOdds Ratio	[95%Conf.Interval]		P value
**Age**								
18–39	1.000				1.000			
**40–61**	**2.705**	**1.074**	**6.816**	**0.035**	2.016	0.707	5.749	0.190
**62–83**	**11.295**	**4.363**	**29.239**	**0.000**	**10.858**	**3.241**	**36.377**	**0.000**
**BMI**								
Underweight (<18.5)	1.000				1.000			
Normal (18.5–25)	1.588	0.584	4.317	0.365	2.550	0.771	8.429	0.125
**Overweight/Obese (> = 25)**	1.912	0.581	6.297	0.286	2.496	0.521	11.959	0.253
**Sex**				** **				** **
Male	1.000				1.000			
**Female**	**0.488**	**0.250**	**0.952**	**0.035**	0.730	0.278	1.915	0.522
**Education**								
Never/Rarely formal education	1.000				1.000			
Primary Education	1.193	0.561	2.536	0.646	1.176	0.438	3.160	0.748
Secondary Education	**0.287**	**0.084**	**0.989**	**0.048**	0.710	0.161	3.118	0.650
Post-Secondary	0.220	0.028	1.695	0.146	1.506	0.098	23.094	0.769
**Internet Access**								
Never/Rarely	1.000				1.000			
Weekly/Daily	0.158	0.021	1.184	0.072	0.142	0.010	1.963	0.145
**Tobacco Use**								
Never/Rarely	1.000				1.000			
**Weekly/Daily**	1.785	0.870	3.663	0.114	2.115	0.765	5.853	0.149
**Smoking**								
Never/Rarely	1.000				1.000			
Weekly/Daily	1.635	0.811	3.295	0.169	1.542	0.636	3.736	0.338
**Alcohol Drinking**								
Never/Rarely	1.000				1.000			
Weekly/Daily	1.416	0.737	2.720	0.297	0.928	0.397	2.169	0.863
**Exercise**								
Never/Rarely	1.000				1.000			
Weekly/Daily	0.606	0.326	1.126	0.113	0.570	0.227	1.430	0.231
**Location**								
Semi urban	1.000				1.000			
Rural	0.560	0.285	1.099	0.092	0.400	0.157	1.023	0.056
**Geography**								
Hills	1.000				1.000			
Terrai	0.749	0.387	1.451	0.392	0.679	0.228	2.022	0.487
**Household size**								
(1–4)	1.000				1.000			
(5–9)	1.546	0.692	3.450	0.288	2.325	0.898	6.015	0.082
(10+)	2.333	0.795	6.846	0.123	0.982	0.252	3.819	0.979
**Milk**								
Never/Rarely	1.000				1.000			
Weekly/Daily	**2.662**	**1.144**	**6.192**	**0.023**	**4.343**	**1.253**	**15.051**	**0.021**
**Yoghurt**								
Never/Rarely	1.000				1.000			
Weekly/Daily	0.996	0.416	2.382	0.992	0.504	0.147	1.729	0.276
**Fermented food**								
Never/Rarely	1.000				1.000			
Weekly/Daily	1.069	0.558	2.049	0.840	1.676	0.750	3.746	0.208
**Cooked food**								
Never/Rarely	1.000				1.000			
Weekly/Daily	0.846	0.428	1.672	0.630	1.037	0.431	2.494	0.936
**Scarcity of food**								
No	1.000				1.000			
Yes	1.872	0.961	3.648	0.066	1.699	0.733	3.939	0.217
**Fish**								
Never/Rarely	1.000				1.000			
Weekly/Daily	0.591	0.172	2.026	0.402	1.025	0.230	4.579	0.974
**Meat**								
Never/Rarely	1.000				1.000			
Weekly/Daily	0.838	0.402	1.750	0.639	0.799	0.310	2.062	0.643
**Constant**					0.025	0.002	0.321	0.005

Systolic hypertension was defined as systolic blood pressure ≥ 140 mmHg. Column 1 includes variables tested for association with systolic hypertension using univariate and multivariable logistic regressions in the 311 study participants from Nepal. Crude odds ratio was derived from the univariate logistic regression and adjusted odds ratio was derived from the multivariable logistic regression. Significant results with p values < 0.05 are shown in bold.

**Table 5:** Factors associated with diastolic hypertension in study participants.

	UNIVARIATE ANALYSIS				MULTIVARIATE ANALYSIS			
	Crude Odds Ratio	[95% Conf.Interval]		P value	Adjusted Odds Ratio	[95% Conf.Interval]		P value
**Age**								
18–39	1.000				1.000			
**40–61**	**2.998**	**1.514**	**5.940**	**0.002**	**2.573**	**1.170**	**5.658**	**0.019**
**62–83**	**4.902**	**2.203**	**10.905**	**0.000**	**5.292**	**1.914**	**14.629**	**0.001**
**BMI**								
Underweight (<18.5)	1.000				1.000			
Normal (18.5–25)	1.648	0.694	3.913	0.257	1.942	0.736	5.125	0.180
**Overweight/Obese (> = 25)**	**3.255**	**1.205**	**8.792**	**0.020**	3.077	0.926	10.228	0.067
**Sex**								
Male	1.000				1.000			
**Female**	**0.408**	**0.231**	**0.721**	**0.002**	0.679	0.310	1.487	0.333
**Education**								
Never/Rarely formal education	1.000				1.000			
Primary Education	1.351	0.700	2.607	0.370	1.318	0.577	3.009	0.512
Secondary Education	0.564	0.245	1.298	0.178	0.836	0.284	2.458	0.744
Post-Secondary	0.307	0.069	1.363	0.120	0.810	0.111	5.919	0.835
**Internet Access**								
Never/Rarely	1.000				1.000			
Weekly/Daily	0.432	0.147	1.269	0.127	0.452	0.101	2.023	0.299
**Tobacco Use**								
Never/Rarely	1.000				1.000			
**Weekly/Daily**	**2.351**	**1.279**	**4.322**	**0.006**	2.112	0.927	4.811	0.075
**Smoking**								
Never/Rarely	1.000				1.000			
Weekly/Daily	1.247	0.672	2.314	0.484	0.803	0.381	1.693	0.564
**Alcohol Drinking**								
Never/Rarely	1.000				1.000			
Weekly/Daily	**2.371**	**1.342**	**4.190**	**0.003**	1.751	0.880	3.485	0.110
**Exercise**								
Never/Rarely	1.000				1.000			
Weekly/Daily	0.650	0.338	1.249	0.196	0.620	0.289	1.328	0.219
**Location**								
Semi urban	1.000				1.000			
Rural	1.202	0.693	2.084	0.512	1.428	0.679	3.002	0.347
**Geography**								
Hills	1.000				1.000			
Terrai	0.625	0.355	1.098	0.102	0.916	0.388	2.163	0.842
**Household size**								
(1–4)	1.000				1.000			
(5–9)	1.346	0.708	2.559	0.365	1.464	0.705	3.040	0.307
(10+)	1.426	0.549	3.706	0.467	0.914	0.282	2.966	0.881
**Milk**								
Never/Rarely	1.000				1.000			
Weekly/Daily	1.721	0.777	3.812	0.181	2.671	0.964	7.399	0.059
**Yoghurt**								
Never/Rarely	1.000				1.000			
Weekly/Daily	0.776	0.357	1.690	0.523	0.478	0.178	1.285	0.143
**Fermented food**								
Never/Rarely	1.000				1.000			
Weekly/Daily	0.919	0.530	1.594	0.763	1.082	0.565	2.069	0.813
**Cooked food**								
Never/Rarely	1.000				1.000			
Weekly/Daily	0.898	0.502	1.605	0.716	0.789	0.373	1.668	0.535
**Scarcity of food**								
No	1.000				1.000			
Yes	1.363	0.785	2.367	0.272	1.311	0.663	2.593	0.437
**Fish**								
Never/Rarely	1.000				1.000			
Weekly/Daily	1.214	0.522	2.827	0.652	1.187	0.399	3.530	0.758
**Meat**								
Never/Rarely	1.000				1.000			
Weekly/Daily	**1.916**	**1.080**	**3.400**	**0.026**	**2.218**	**1.066**	**4.614**	**0.033**
Constant					0.038	0.005	0.274	0.001

Diastolic hypertension was defined as diastolic blood pressure ≥ 90 mmHg. Column 1 includes variables tested for association with systolic hypertension using univariate and multivariable logistic regressions in the 311 study participants from Nepal. Crude odds ratio was derived from the univariate logistic regression and adjusted odds ratio was derived from the multivariable logistic regression. Significant results with p values < 0.05 are shown in bold.

### Distribution of risk factors among the hypertensive individuals

Several modifiable risk factors such as high sodium intake, low potassium intake, alcohol consumption, smoking, overweightness/obesity, lack of physical activity, and unhealthy diet have been established as determinants of hypertension [20]. Therefore, to evaluate whether previously established risk factors contribute to hypertension in these indigenous Nepali populations, we assessed the distribution of five determinants in these individuals, namely alcohol consumption, diet with low vegetables, physical activity, overweightness/obesity, and smoking (Table 6). Overall, 30.9% of participants had none of the determinants while 39.9%, 21.2%, 8.0% and 1.0% had 1, 2, 3, and 4 determinants respectively but none of the participants had all 5 risk factors. Among the hypertensive individuals, 43.2% had 1 determinant, 32.4% had 2 determinants, 10.8% had 3 determinants, and none of them had more than 3 determinants. 13.5% of the hypertensive individuals had none of these previously identified determinants (Table 6). Since most of these determinants were not individually associated with hypertension in our participants (Table 3), we evaluated whether they collectively contribute to hypertension in these populations by counting the total number of determinants in each participant. We found that including the determinant counts significantly improves the fit of the model compared to a null model with no variables (P = 0.0021, *Wald test*). Moreover, relative to the individuals with none of the determinants, those that had 1 or more previously established determinants were more likely to have hypertension (OR > 2, P < 0.05, Table 6). This model explained an additional ~2.8% of the variance in hypertension in these participants. Together, these results indicate that the previously established hypertension risk factors do contribute to hypertension in indigenous Nepali populations but their effect sizes are smaller compared to the general Nepali population.

**Table 6:** Percent distribution of established determinants in study participants.

No. of Risk Factors	All (n = 311)	Non-hypertensive (n = 237)	Hypertensive (n = 74)	Odds Ratio (OR)	[95% CI]		P-values
None	29.9%	35.0%	13.5%	1.000			
1	39.9%	38.8%	43.2%	2.887	1.337	6.232	0.007
2	21.2%	17.7%	32.4%	4.743	2.077	10.831	0.000
3	8.0%	7.2%	10.8%	3.32*	1.162*	9.488*	0.025*
4	1.0%	1.3%	0.0%	--	--	--	--
5	0.0%	0.0%	0.0%	--	--	--	--
Constant				0.120	0.063	0.232	0.000

Percent distribution of five previously established hypertension determinants in the study. A logistic regression was used to evaluate whether increasing numbers of risk factors (1, 2, or ≥3) are associated with elevated levels of hypertension in the study populations. The “*” indicates individuals were grouped into a single category due to limited number of individuals with ≥3 risk factors for the statistical analysis.

## Discussion

Previous studies examining the prevalence of NCDs using nationally representative datasets such as the Nepal Demographic and Health Survey 2016 (NDHS) have identified high burden of hypertension in Nepal [13, 21]. However, NHDS does not disclose the specific population groups and their sample sizes, which makes it difficult to determine the prevalence of NCDs in a particular population group within Nepal. By focusing on specific population groups, this study aimed to provide a finer understanding of prevalence of NCDs in specific communities within Nepal. The two major goals of this study were to assess the prevalence of NCDs and identify their determinants in the indigenous populations of Nepal that have been underrepresented in previous reports. Our results revealed an appreciable burden of NCDs in indigenous Nepali populations. We detected obesity and elevated heart rate at low abundance but about half of the participants (52%) had elevated blood pressure (SBP ≥ 130mmHg or DBP ≥ 80mmHg) and a quarter of participants presented with hypertension (SBP ≥ 140mmHg and/or DBP ≥ 80mmHg). This finding is consistent with previous studies that have reported a high overall prevalence of hypertension in the general Nepali population [13] as well as in the neighboring regions [22, 23], indicating that hypertension is a major threat in South Asia that needs serious consideration at the national as well as the local levels. This is likely only the tip of the iceberg because additional chronic diseases such as diabetes, cardiovascular diseases, cancer, etc., likely exist in these populations but remain undetected.

Given the prevalence of hypertension in our dataset, we evaluated the link between hypertension and 19 variables, which included previously established determinants of hypertension such as sex, age, BMI, education, alcohol and tobacco use, physical inactivity, and processed diet that are rich in sodium or carbohydrates and low in fiber content. Many of our results are consistent with findings from previous studies. For example, in our univariate analysis, we found hypertension was more prevalent in indigenous men, which corroborates previous reports [23, 24]. The lower rates of hypertension as well as lowered systolic and diastolic blood pressures in women may be likely due to the vasorelaxation properties of estrogen [24–26]. Multivariable analysis revealed strong positive associations between age and BMI with hypertension, which is also consistent with previous studies [20, 27]. However, many of the factors such as education, internet access, geographical location (rural vs urban), smoking, alcohol consumption, and physical activity, that have been previously established as determinants of hypertension in the general Nepali [16, 24, 28] as well as worldwide populations [20] were not individually associated with hypertension in the indigenous participants in this study. The lack of association between previously established determinants of hypertension in this study could be due to insufficient statistical power resulting from the small sample size, although other studies with similar sample sizes have been able to link the established determinants with hypertension in non-indigenous Nepali populations [22, 23, 29, 30].

The weak associations of previously established hypertension determinants in this study population may reflect the lifestyle differences between the indigenous and the general Nepali population. For example, most indigenous populations reside in rural or peri-urban areas of Nepal, have little to no formal education, and lead a highly active agrarian life. All of the participants in this study resided in rural or semi urban areas, 73% were not educated beyond the primary level, and 81% were physically active. Therefore, geographical location, education, or physical activities are not major contributors to hypertension in these populations. Furthermore, the consumption of processed foods with high sodium or carbohydrate content is one of the major determinants of hypertension. Although our surveys did not include sodium or potassium intake of our participants, we found that processed food is not likely contributing to hypertension in these populations. A majority (68%) of participants ate home-cooked meals low in animal protein, 97% reported that diet consisted of fresh vegetables twice a day, and 49% reported supplementing their diet with fermented foods daily or weekly. These findings are consistent with previous reports that reported high vegetable consumption in Nepal [19] and lower consumption of processed foods in rural Nepali populations due to financial constrains [13]. Similarly, smoking was not common in these populations, 75% of participants did not smoke, and many of those who smoked were occasional smokers, likely because cigarettes are not always affordable. However, non-smoking tobacco use is common among the lowest socio-economic groups within Nepal [31]. Almost a quarter (21.5%) of our participants consumed non-smoking tobacco products regularly, which was significantly associated with hypertension in this study. A vast majority of the non-smoking tobacco users in our dataset (85%) were male, which could partly explain the elevated prevalence of hypertension in indigenous Nepali males.

To further assess whether the previously established determinants contributed to hypertension in our study participants, we assessed the link between number of risk factors at the individual level and hypertension risk. This analysis further revealed that the collective contributions of these determinants are detectable in these populations. Individuals with one or more of these factors were more likely to have hypertension compared to the individuals with none of these factors. This finding indicates that the globally relevant determinants also affect hypertension in the Nepali indigenous populations but their individual contributions are smaller in these indigenous peoples compared to the general Nepali populations. Finally, we found that 13.5% of the hypertensive individuals in this study had none of the commonly known risk factors for hypertension. Furthermore, a multivariable regression model including all of the variables in our dataset was able to explain only ~14% variance in hypertension, indicating that additional determinants of hypertension in these populations remain unidentified. It is possible that the determinants of hypertension in these populations may include population specific cultural, dietary, lifestyle elements, which may not have been captured by our survey questionnaire despite our best effort to capture elements of traditional Nepali lifestyle (e.g., fermented food consumption). For instance, it was difficult to measure participants’ salt intake, which is a known determinant of hypertension. Identification of additional hypertension determinants may require future studies to incorporate population specific cultural elements in addition to the generalized questions included in the standardized survey questionnaires.

We focused on indigenous populations because they are underrepresented in nationally representative surveys and hospital-based public health research. Therefore, they are the highest risk groups of developing and dying from NCDs. Our study does not encompass all of the indigenous populations of Nepal, nor does it assess all of the NCDs. Despite these limitations, our study highlights that hypertension is prevalent in indigenous Nepali populations, and in addition to the previously established determinants that contribute to hypertension in the general Nepali population, novel hypertension associated risk factors likely exist in these populations but remain to be identified. Thus, intervention strategies developed for the general population may not be sufficient to address the growing burden of NCDs in indigenous Nepali peoples. Larger and more comprehensive future studies are needed to detect the array of NCDs and pinpoint their determinants in the indigenous peoples. As such, we may need to observe NCDs using a different lens when it comes to indigenous peoples.

## Supporting information

S1 Table Survey questions and the codes used to represent them in the dataset. (DOCX)

S2 Table Individual responses to the survey questions. (CSV)

## References

[1] Heller O, Somerville C, Suggs LS, Lachat S, Piper J, Aya Pastrana N, et al. The process of prioritization of non-communicable diseases in the global health policy arena. Health policy and planning. 2019;34(5):370–83. doi: 10.1093/heapol/czz043 31199439 PMC6736081

[2] Bigna JJ, Noubiap JJ. The rising burden of non-communicable diseases in sub-Saharan Africa. The Lancet Global Health. 2019;7(10):e1295–e6. doi: 10.1016/S2214-109X(19)30370-5 31537347

[3] Allen LN, Pullar J, Wickramasinghe KK, Williams J, Roberts N, Mikkelsen B, et al. Evaluation of research on interventions aligned to WHO ‘Best Buys’ for NCDs in low-income and lower-middle-income countries: a systematic review from 1990 to 2015. BMJ global health. 2018;3(1). doi: 10.1136/bmjgh-2017-000535 29527342 PMC5841523

[4] Allotey P, Davey T, Reidpath DD. NCDs in low and middle-income countries-assessing the capacity of health systems to respond to population needs. BMC public health. 2014;14(2):1–3. doi: 10.1186/1471-2458-14-S2-S1 25082328 PMC4120152

[5] Kankeu HT, Saksena P, Xu K, Evans DB. The financial burden from non-communicable diseases in low-and middle-income countries: a literature review. Health Research Policy and Systems. 2013;11(1):1–12. doi: 10.1186/1478-4505-11-31 23947294 PMC3751656

[6] Flood D, Rohloff P. Indigenous languages and global health. The Lancet Global Health. 2018;6(2):e134–e5. doi: 10.1016/S2214-109X(17)30493-X 29389530

[7] Hernández A, Ruano AL, Marchal B, San Sebastián M, Flores W. Engaging with complexity to improve the health of indigenous people: a call for the use of systems thinking to tackle health inequity. International Journal for Equity in Health. 2017;16(1):1–5. doi: 10.1186/s12939-016-0499-1 28219429 PMC5319053

[8] Whitinui P, McIvor O, Robertson B, Morcom L, Cashman K, Arbon V. The World Indigenous Research Alliance (WIRA): Mediating and Mobilizing Indigenous Peoples’ Educational Knowledge and Aspirations. education policy analysis archives. 2015;23(120):n120.

[9] National Foundation for Development of Indigenous Nationalities (NFDIN): an introduction. Lalitapura: NFDIN, Kathmandu, Nepal, 2003.

[10] Bhattachan YK. Consultation and Participation of Indigenous Peoples in Decision-making in Nepal. First Edition ed. Sarah Webster OG, editor. Kathmandu, Nepal: Hisi Offset Printers Pvt. Ltd; 2005 December, 2005. 78–108 p.

[11] Aryal KK, Mehata S, Neupane S, Vaidya A, Dhimal M, Dhakal P, et al. The burden and determinants of non communicable diseases risk factors in Nepal: findings from a nationwide STEPS survey. PloS one. 2015;10(8):e0134834. doi: 10.1371/journal.pone.0134834 26244512 PMC4526223

[12] Mehata S, Shrestha N, Mehta R, Vaidya A, Rawal LB, Bhattarai N, et al. Prevalence, awareness, treatment and control of hypertension in Nepal: data from nationally representative population-based cross-sectional study. Journal of hypertension. 2018;36(8):1680–8. doi: 10.1097/HJH.0000000000001745 29621067

[13] Mishra SR, Ghimire S, Shrestha N, Shrestha A, Virani SS. Socio-economic inequalities in hypertension burden and cascade of services: nationwide cross-sectional study in Nepal. Journal of human hypertension. 2019;33(8):613–25. doi: 10.1038/s41371-019-0165-3 30659279

[14] Shrestha N, Mishra SR, Ghimire S, Gyawali B, Pradhan PMS, Schwarz D. Application of single-level and multi-level modeling approach to examine geographic and socioeconomic variation in underweight, overweight and obesity in Nepal: findings from NDHS 2016. Scientific reports. 2020;10(1):1–14. doi: 10.1038/s41598-019-56847-4 32051421 PMC7016110

[15] Shrestha N, Mishra SR, Ghimire S, Gyawali B, Mehata S. Burden of diabetes and prediabetes in Nepal: A systematic review and meta-analysis. Diabetes Therapy. 2020:1–12. doi: 10.1007/s13300-020-00884-0 32712902 PMC7434818

[16] Bista B, Dhungana RR, Chalise B, Pandey AR. Prevalence and determinants of non-communicable diseases risk factors among reproductive aged women of Nepal: Results from Nepal Demographic Health Survey 2016. PloS one. 2020;15(3):e0218840. doi: 10.1371/journal.pone.0218840 32176883 PMC7075700

[17] Bista DB. People of Nepal. Kathmandu, Nepal: Ratna Pustak Bhandar; 2004.

[18] National population and housing census 2011. Kathmandu,Nepal: National Planning Commission Secretariat Central Bureau of Statistics, 2012.

[19] Nepali S, Rijal A, Olsen MH, McLachlan CS, Kallestrup P, Neupane D. Factors affecting the fruit and vegetable intake in Nepal and its association with history of self-reported major cardiovascular events. BMC Cardiovascular Disorders. 2020;20(1):1–10. doi: 10.1186/s12872-019-01312-3 32972356 PMC7517612

[20] Mills KT, Stefanescu A, He J. The global epidemiology of hypertension. Nature Reviews Nephrology. 2020;16(4):223–37. doi: 10.1038/s41581-019-0244-2 32024986 PMC7998524

[21] Rauniyar SK, Rahman MM, Rahman MS, Abe SK, Nomura S, Shibuya K. Inequalities and risk factors analysis in prevalence and management of hypertension in India and Nepal: a national and subnational study. BMC public health. 2020;20(1):1–11. doi: 10.1186/s12889-019-7969-5 32883278 PMC7469349

[22] Dhungana RR, Devkota S, Khanal MK, Gurung Y, Giri RK, Parajuli RK, et al. Prevalence of cardiovascular health risk behaviors in a remote rural community of Sindhuli district, Nepal. BMC cardiovascular disorders. 2014;14(1):1–8. 25066117 10.1186/1471-2261-14-92 PMC4115072

[23] Neupane D, McLachlan CS, Sharma R, Gyawali B, Khanal V, Mishra SR, et al. Prevalence of hypertension in member countries of South Asian Association for Regional Cooperation (SAARC): systematic review and meta-analysis. Medicine. 2014;93(13). doi: 10.1097/MD.0000000000000074 25233326 PMC4616265

[24] Neupane D, Shrestha A, Mishra SR, Bloch J, Christensen B, McLachlan CS, et al. Awareness, prevalence, treatment, and control of hypertension in western Nepal. American journal of hypertension. 2017;30(9):907–13. doi: 10.1093/ajh/hpx074 28531244

[25] Ghosh S, Mukhopadhyay S, Barik A. Sex differences in the risk profile of hypertension: a cross-sectional study. BMJ open. 2016;6(7). doi: 10.1136/bmjopen-2015-010085 27466234 PMC4964242

[26] Orshal JM, Khalil RA. Gender, sex hormones, and vascular tone. American Journal of Physiology-Regulatory, Integrative and Comparative Physiology. 2004;286(2):R233–R49. doi: 10.1152/ajpregu.00338.2003 14707008

[27] Hasan M, Sutradhar I, Akter T, Das Gupta R, Joshi H, Haider MR, et al. Prevalence and determinants of hypertension among adult population in Nepal: Data from Nepal Demographic and Health Survey 2016. PloS one. 2018;13(5):e0198028. doi: 10.1371/journal.pone.0198028 29852006 PMC5978874

[28] Chataut J, Adhikari R, Sinha N. Prevalence and risk factors for hypertension in adults living in central development region of Nepal. Kathmandu University Medical Journal. 2011;9(1):13–8. doi: 10.3126/kumj.v9i1.6255 22610802

[29] Dhungana RR, Pandey AR, Shrestha N. Trends in the Prevalence, Awareness, Treatment, and Control of Hypertension in Nepal between 2000 and 2025: A Systematic Review and Meta-Analysis. International journal of hypertension. 2021;2021. 10.1155/2021/6610649 PMC7952181 33747559

[30] Khanal MK, Ahmed MM, Moniruzzaman M, Banik PC, Dhungana RR, Bhandari P, et al. Prevalence and clustering of cardiovascular disease risk factors in rural Nepalese population aged 40–80 years. BMC public health. 2018;18(1):1–13. doi: 10.1186/s12889-018-5600-9 29855293 PMC5984400

[31] Shrestha N, Mehata S, Pradhan PMS, Joshi D, Mishra SR. A nationally representative study on socio-demographic and geographic correlates, and trends in tobacco use in Nepal. Scientific reports. 2019;9(1):1–11. doi: 10.1038/s41598-018-37186-2 30804493 PMC6389978

