# Supplementary material for: Prevalence and determinants of hypertension in underrepresented indigenous populations of Nepal
Source: PLOS Glob Public Health. 2022 Feb 18;2(2):e0000133. doi: 10.1371/journal.pgph.0000133 (PMC10021878; doi:10.1371/journal.pgph.0000133)
Supplement: S1 Table — (DOCX) [file pgph.0000133.s001.docx]

| **Questions** | **Codes** |
| --- | --- |
| **A. Basic information** |  |
| Patient ID | PID |
| Age | AGE |
| Sex | SEX |
| Household size | HS |
| Location status | LOC |
| **D. Phenotypes** |  |
| Height (cm) | HGT |
| Weight (kg) | WGT |
| Blood Pressure (systolic mmHg) | SYS |
| Blood Pressure (diastolic mmHg) | DIA |
| Pulse (bpm) | BPM |
| **E. Household characteristics** |  |
| Do you have internet in your household? | INT |
| If yes, how do you connect to the Internet? | INT_MOD |
| **F. Dietary information** |  |
| What type of fruits and vegetables do you eat? | VEG |
| How much fruits and vegetables do you eat? | VEG_FREQ |
| How much fish do you eat? | FISH |
| How much meat do you eat? | MEAT |
| What do you drink on a regular basis? Milk | MILK |
| Do you eat yogurt? | YGRT |
| How often do you eat yogurt? | YGRT_FREQ |
| Do you eat fermented food like gundruk, sinki or *khadeko achar*? | FERM |
| How often do you eat such fermented food? | FERM_FREQ |
| If yes, how often do you cook your food? | COOK_FREQ |
| Is there scarcity of food in your household? | SCAR |
| **G. General Health information** |  |
| Do you smoke? | SMOK |
| If yes, how many cigarettes do you smoke daily? | SMOK_QTY |
| Do you use tobacco products other than cigarettes? | TOBA |
| Do your drink alcohol? | ALCO |
| If yes, how often do you drink? | ALCO_FREQ |
| How much do you drink in one sitting? | ALCO_QTY |
| Do you exercise or do any kind of physical activity? | EXER |
| If yes, how often do you exercise or do any kind of physical activity? | EXER_FREQ |

**S1 Table:** Questions asked in the survey and the codes used to represent

them in the dataset (found in S2 table)
